# Diseases of the Nervous System

**Published:** 1917-07

**Authors:** 


**Diseases of the Nervous System.** By H. CAMPBELL THOMSON, M.D., F.R.C.P. Second Edition, revised and enlarged. Cassell & Co. Ltd., London, New York, etc. 1915.

Of the smaller text-books on nervous diseases this is one of the best, and students and practitioners have found that they have in it a useful guide to this difficult branch of medicine. A second edition has been called for, the original work has therefore been revised and enlarged, and in part remodelled. Thus the recent investigations into Syphilis of the Nervous System have necessitated a reconstruction of this section. New chapters on the General Functions of the Brain, on the applications of the methods of Experimental Psychology to Clinical Work, and on the Sympathetic System have been added. The book retains the features of clearness, accuracy, and brevity which gained for it a favourable reception on its first appearance. and it has been extended and brought thoroughly up to date, so that it may be taken as a trustworthy guide. There are many good illustrations. It has always seemed to us that in the smaller manuals or text-books the very rare diseases might be left out, as from exigencies of space the descriptions are of necessity too brief to aid the practitioner to recognise them. For diseases of which the ordinary man will probably never see a case or only one in a life-time, he might well be referred to the large treatises.

